# Latent profiles and transitions of social isolation among older adults and their influencing factors: a person-centered approach

**DOI:** 10.3389/fpubh.2025.1475951

**Published:** 2025-04-10

**Authors:** Xuezhi Liu, Yanzhen Zhang, Jianxiao Wu, Yadi Zeng, Lingjing Guo, Baojuan Ye

**Affiliations:** ^1^School of Education, School of Psychology, Jiangxi Normal University, Nanchang, China; ^2^Jiangxi College of Foreign Studies, Nanchang, China; ^3^Donald Bren School of Information & Computer Science, University of California, Irvine, CA, United States; ^4^Nanchang Institute of Technology, Nanchang, China; ^5^School of Psychology, Shanxi Normal University, Xian, China; ^6^Mental Health Education Center, Chengdu University, Chengdu, China

**Keywords:** older people, social isolation, latent profile analysis, latent transition analysis, longitudinal study

## Abstract

**Introduction:**

In the context of global aging, it is crucial to understand the heterogeneity, changing trends, and influencing factors of social isolation in older people.

**Methods:**

7198 older people over 60 in China Longitudinal Aging Social Survey in 2016 and 2018 were analyzed, using Latent Profile Analysis, Latent Transition Analysis, and multiple logistic regression analysis.

**Results:**

The study identified five categories of social isolation among older people; the rural, widowed, with low daily living activity ability, and with low cognitive ability were more likely to belong to the social isolation category; the stability of various profiles of social isolation was strong, but the high-level social network category had the danger of changing to the medium-level social network category and the social isolation risk category; over time, those with high daily living activity ability moved into worse social isolation categories, while those with high cognitive ability moved into better categories.

**Discussion:**

The social isolation status of the older adult had group heterogeneity and transitioned over time. The findings provide empirical evidence for taking targeted measures based on the influencing factors in older people to achieve successful aging.

## 1 Introduction

1/6 people in the world will be over 60 years old by 2030 according to the World Health Organization (1). From 2020 to 2030, the number of people over 60 will increase to 1.4 billion from 1 billion. China also faces the major challenge of aging, because China has the largest older adult population in the world (2, 3). Factors such as declining fertility rates, longer lifespans, and the aging of the baby boom generation have led to an aging society (4, 5), while unprepared infrastructure such as inadequate medical facilities, poor transportation, and inadequate housing have exacerbated the challenges posed by aging (6). Although everyone is going to die, successful aging is important for alleviating the social and economic pressures of an aging population (7). Social relationships are important for both physical health (8, 9) and mental health (10, 11). They are the key factors affecting successful aging (12). Social isolation, an objective and quantifiable reflection of poor social relationships, means the reduction in social networks and lacking social connections, such as living alone, having few friends or family, and limited contact with people (13). It affects physical function, cognitive function, and mental health in older adults (14) and is associated with increased mortality (15). A study on the aged population in urban and rural China, which included 220,506 participants, found a prevalence of 36.6% of loneliness among older Chinese individuals (16). Therefore, it is important to pay attention to the change in the social isolation status of older people and then adopt targeted prevention and intervention measures to improve the quality of life and promote the successful aging of older people (17).

### 1.1 Heterogeneity and transition trajectory of social isolation

Magnusson and Mahoney (18) emphasize the Holistic-interaction Perspective and think that the existence of individual differences does not mean that development is disordered, and individuals with differences will partly move in different directions in the following development. Over time, more stable “types” will eventually emerge, that is, in the multi-dimensional space of psychological and behavioral development. Individuals who are brought together by a similar combination of psychological and behavioral characteristics show clearer homogeneity within the individual categories and clearer differentiation between the individual categories. Some studies have found that many interventions designed to solve the problem of social isolation in older people are not effective; an important reason is the heterogeneity of the older people with social isolation (19). Fortunately, the categorization of social isolation in the older adults has received increasing attention in Western nations. Previously, most studies classified social isolation status based on the total scores or dimension- specific scores from standardized scales, overlooking individual item responses and failing to dig into the data (20).

According to the developmental situation theory, individual development has plasticity, and individuals are affected by situational events constantly and keep continuous development and change (21). The social relationship network of older people may change with time. These observations suggest that older adults who previously belonged to one subgroup of social networks may shift to another subgroup because of changes in certain indicators. Studies have found that older people in different social relationship networks or loneliness subgroups have different transformation or stability patterns within a certain time (22, 23).

Moreover, factors such as internet penetration rate (24), social and cultural background (e.g., collectivism vs. individualism) (25), and other regional influences contribute to differences in social isolation status among older people across countries and regions (26, 27). However, research on these dynamics in China is lacking. The Chinese senior population is a unique situation that needs more research because of its particular cultural and social environment, quickly shifting demography, and rapid use of technology. Furthermore, even though many studies have explored the effects of social isolation of the older adults on depression, mortality, physical health status, and other factors (8, 28, 29), in-depth studies on the heterogeneity of social isolation of the older adults are rare, especially the large sample longitudinal tracking literature on the heterogeneity of social isolation of the older adults in China. Critical questions remain unanswered, including whether the patterns of social isolation among Chinese seniors are stable, whether they follow a unique development trajectory, and what factors affect these trajectories.

### 1.2 Influencing factors of heterogeneity and transition trajectory of social isolation

Results from cross-sectional data show that social isolation has significant differences in demographic variables (30, 31). Both males and females are strongly influenced by structural systems with different opportunities, demands, and constraints, which shape different social relationships (32). However, the effects of gender on social isolation are inconsistent (32–34); socioeconomic status (SES) determines the opportunities and ability of older persons to maintain and utilize non-family relationships (35). The influence of residence and spouse status on social isolation is more consistent: rural older people and older people without spouses are more at risk of social isolation (23, 36, 37). Recently, Sung et al. (23) explored the factors related to the change in social network types of older people and found that older, less educated, and worsening functional and mental health older people were more likely to transition into less diverse types compared with remaining in the same type. In addition, cognitive ability, and daily living activity ability in older people are closely related to social isolation (12, 38), but their influence on the transition trend of social isolation status is still lacking.

### 1.3 This study

To explore the categories of social isolation among the Chinese older adults and the transition trends between these categories over time, we adopted person-centered approaches utilizing Latent Profile Analysis (LPA) and Latent Transition Analysis (LTA). In recent years, person-centered research methodologies have gained prominence by identifying similar individuals with similar feature patterns to capture diverse behavioral patterns. The aim is to trace group heterogeneity and elucidate the association between explicit variables through independent latent variables categorized by type, thereby revealing differences among individuals or developmental stages (39). Compared to traditional mean segmentation or cluster analysis, LPA offers greater accuracy, objectivity, and well-fitted models (40). LTA has advantages in capturing dynamic changes, estimating transition probabilities, flexible modeling, and supporting evaluation of intervention effects, and so on (41, 42). Given these benefits, LPA and LTA are well-suited for testing our research hypotheses.

Based on Holistic interaction Perspective, developmental situation theory and previous studies, this study proposed the following research hypotheses:

*H*1: There are different latent profiles of social isolation among the older adults.

*H*2: Individuals in the social isolation categories of the older adults will transition to other categories over time.

*H*3: The categories of social isolation of the older adults and the transition among categories are affected by various factors.

## 2 Methods

### 2.1 Study sample

The data of this study was secondary data from the China Longitudinal Aging Social Survey (CLASS), which adopted a stratified and multi-stage probability sampling method. County-level regions (including counties, county-level cities, and districts) were selected as the primary sampling units, and then village/neighborhood committees were selected as the secondary sampling units. The respondents were Chinese citizens over the age of 60 from these units. The project covers 476 villages/neighborhood committees in 30 provinces/autonomous regions/municipalities across China. The survey began in 2011, followed by the first nationwide baseline survey in 2014 and two follow-up surveys in 2016 and 2018. This study selected the data from the survey in 2016 and 2018 for several reasons: first, the data in 2016 and 2018 were more contemporary than the data in 2014; second, Latent Transition Analysis (LTA) could be completed by using two waves of data; third, after matching the three waves of data, only 3,852 cases could be retained, resulting in data loss; fourth, the follow-up survey in 2016 and 2018 had further adjusted and enriched the questionnaire content on the basis of 2014, which meant that the survey content in 2016 and 2018 was more stable and consistent. A total of 11,471 data were collected in 2016, and 11,419 data were collected in 2018. Each participant was given a unique ID, which was used to match data from 2016 and 2018. We removed cases in which participants only participated in 2016 or 2018: 1,830 cases in 2016 and 1,778 cases in 2018. Also, 2,443 cases were excluded due to too many missing values on key variables, regular responses, or logical errors. Finally, the data of 7,198 older people over 60 years old was kept. Descriptive statistics for the sample are presented in Table 1.

**Table 1:** Descriptive statistics at T1 (2016).

Demographic variables	N	Percentage	M	SD
Gender				
Male	3,807	52.89%		
Female	3,391	47.11%		
Household registration				
Rural	3,786	52.60%		
Urban	3,412	47.40%		
Marital status				
Widowed	1,263	17.55%		
Non-widowed	5,935	82.45%		
Age			68.90	6.86
Daily living activity ability			2.94	0.16
Cognitive ability			13.69	2.81

### 2.2 Measures

#### 2.2.1 Social isolation

The simplified Lubben Social Network Scale (LSNS-6) revised by Chang et al. was utilized (43–45). There were six items on the scale, divided into two dimensions: family network (3 questions) and friend network (3 questions) and were scored for six points (0 = none, 1 = 1, 2 = 2 and more, 3 = 3–4, 4 = 5–8, 5 = 9 and more). The scale has been widely used with good reliability and validity (46, 47). In CLASS, the Cronbach’s *α* coefficient of this scale in 2016 and 2018 were 0.871 and 0.870, respectively.

#### 2.2.2 Daily living activity ability

The scale developed by Lawton et al. (48) was utilized, including two subscales of basic life activity ability and instrumental daily activity ability. The basic activities included dressing, bathing, eating, taking medicine, incontinence, going to the toilet, and walking indoors; instrumental daily activities included making phone calls, taking medicine, taking transportation, shopping, cooking, and doing housework. The scale was scored on three points (1 = needn’t help from others, 2 = need some help, 3 = no ability at all). All items were scored in reverse. Cronbach’s *α* coefficient of this scale in 2016 and 2018 in CLASS was 0.889 and 0.918, respectively.

#### 2.2.3 Cognitive ability

Referring to the Minimum Mental State Examination, MMSE (49), 16 items of the scale were divided into 4 dimensions: orientation (five questions), memory ability (3 questions), attention and calculation ability (five questions), and recall ability (3 questions). Wrong answers were worth 0 points, and correct answers were worth 1 point. The total score was 0–16 points. The scale was widely used in testing global cognitive function in research settings and clinical screening (50). Cronbach’s *α* coefficient of this scale in 2016 and 2018 in CLASS was 0.822 and 0.817, respectively.

### 2.3 Data processing

SPSS 26.0 was used for data entry and descriptive statistical analysis of sociodemographic characteristics, including the mean, standard deviation, and percentages. Mplus 7 was used for latent profile analysis (LPA), latent transition analysis (LTA), and multiple logistic regression analysis. We used LPA to classify social isolation at two time points, evaluating fit using AIC, BIC, sample size-adjusted BIC (aBIC), Entropy index, likelihood ratio test index LMR-LRT (*p*-value), and Bootstrap-based likelihood ratio test BLRT (*p*-value) index. The smaller the three information evaluation indicators (AIC, BIC, and aBIC), the better the model fit. The value range of the Entropy index was 0 to 1, and the closer it was to 1, the more accurate the classification was. When the Entropy index was less than 0.60, it meant that more than 20% of individuals had a classification error. When the Entropy index reached 0.8, it indicated that the classification accuracy exceeded 90% (51). When the *p*-values of LMR-LRT and BLRT reached a significant level, it indicated that the model with k categories was significantly better than that with k-1 categories (52, 53).

LTA was used to calculate the probability of change for different latent profiles over time. Based on LPA, LTA without covariates was used to investigate the changing relationship between various social insolation categories at two time points. The classification results of the two-time points obtained by the LTA were not the same as the classification results of the LPA exactly at two-time points. In the LPA, the classification criteria of the two-time points were determined separately to make the fitting function of the respective model reach the minimum value. However, the category means, and variance of each dimension must be set equal across time points in LTA, so that the classes of the two-time points are comparable (53). To ensure that the five groups at the two-time points maintain the original LPA classification characteristics when running LTA, we took the LPA analysis results as the parameter setting values for LTA.

Multiple logistic regression analysis was used to explore the impact of demographic variables, daily living activity ability, and cognitive ability on the grouping of social isolation of older people. The results were dominated by the value of OR (Odd Ratio), which referred to the probability of cases belonging to the other group compared with the reference group when two groups were compared under the influence of covariates (20).

The cases that remained in the original category from T1 to T2 were taken as the reference category. The OR referred to the change ratio between the probability of the cases changing to other categories and the probability of staying in the same category now. If the OR was greater than 1, it meant that under the influence of covariates, the probability of the transition increased, and vice versa, it decreased (54, 55). We included all relevant demographic characteristics as covariates in the same model to simultaneously adjust for potential confounding effects of demographic variables.

## 3 Results

### 3.1 Latent profiles of social isolation of the older people

We independently performed exploratory cross-sectional LPA with 1 to 6 profile solutions for two time points. At T1 and T2, the fit indices AIC, BIC, and aBIC for the classification of social isolation of the older people were monotonically decreasing, and the values of Entropy were all greater than 0.8. LMR-LRT and BLRT were also significant. According to these indicators synthetically, social isolation was best divided into five categories. First, Entropy was the largest value when divided into five categories. Secondly, although AIC, BIC, and aBIC have been monotonically decreasing, their speed slowed down significantly in the five categories of models. Finally, when the older people were divided into five categories, the category of friend isolation features appeared. It conformed to the classification of social isolation experience because friend isolation was a particularly important subtype of social isolation for older people (36, 56).

**Figure 1:**
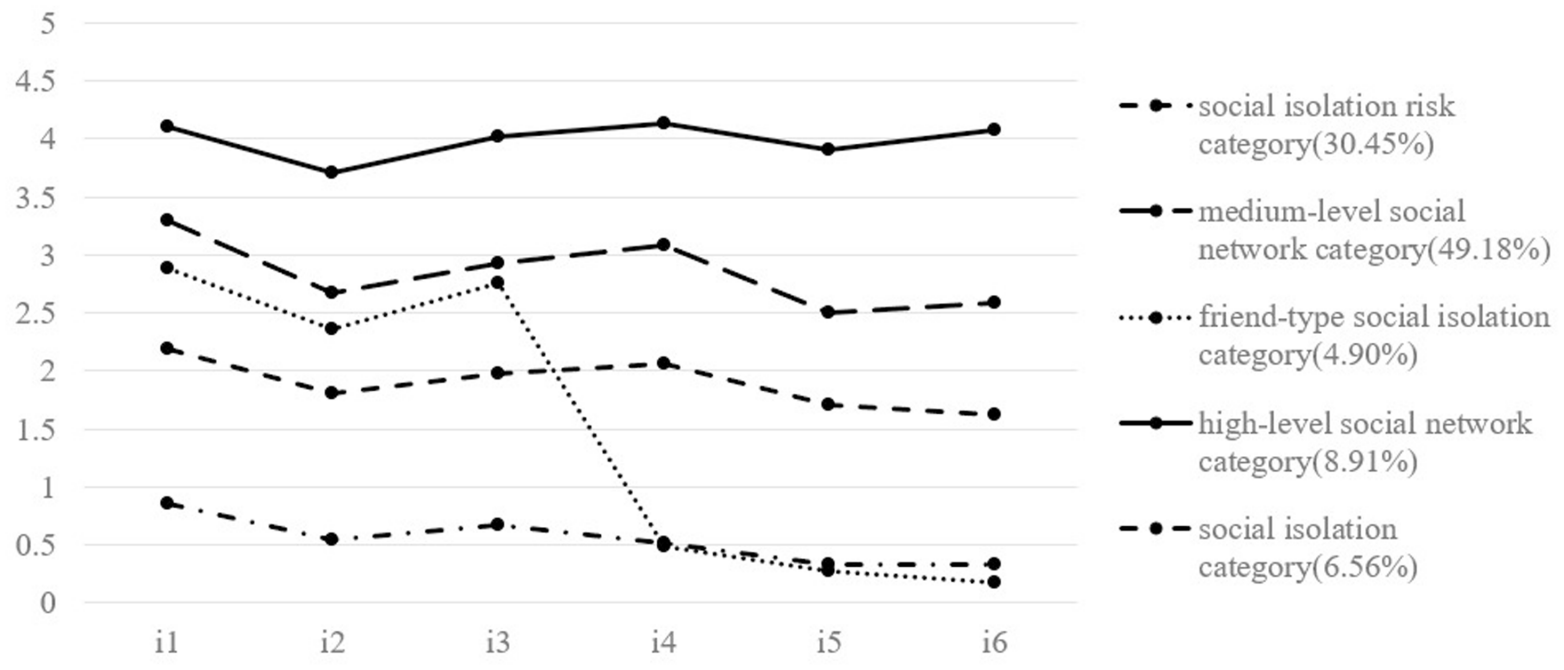
LPA results at T1 (2016). i1-i3 were the three items of the family network dimension of the social network scale, and i4-i6 are the three items of the friend network dimension of the social network scale. The percentage represents the proportion of different categories of older adult in all participants.

**Figure 2:**
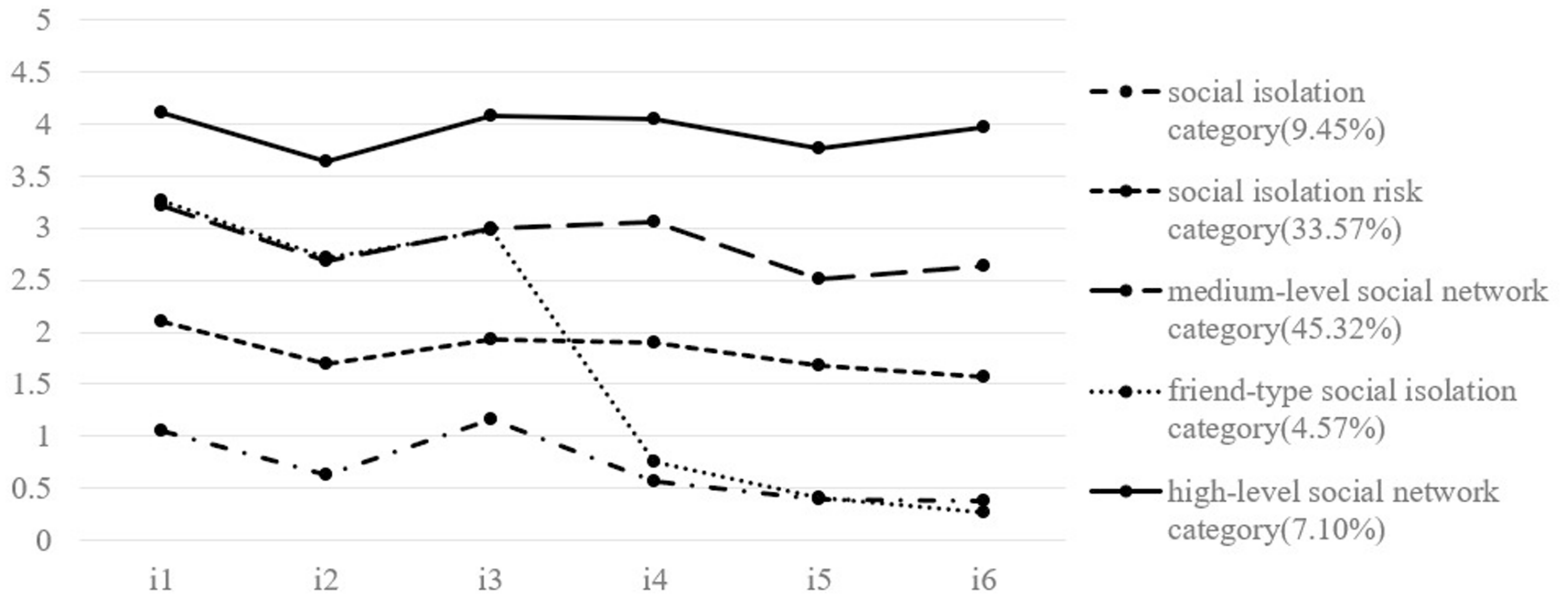
LPA results at T2 (2018). i1-i3 were the three items of the family network dimension of the social network scale, and i4-i6 are the three items of the friend network dimension of the social network scale. The percentage represents the proportion of different categories of older adult in all participants.

We named the five categories according to their characteristics in the item scores of social isolations. As shown in Figures 1, 2, the horizontal axis represented the 6 items of the Social Network Scale, and the vertical axis represented the scores on 6 items. There were four lines with roughly the same trend of balanced scores in all items from bottom to top and a special line with unbalanced scores. The first one was the bottom line, which represented the first category. In this line, the score of each item was less than 1 point, indicating that older people in this category have almost no family or friends to interact with in contacting, talking about personal affairs, or seeking help. So, we named it the social isolation category (SIC, 6.56 and 9.45% at two time points). The second line from the bottom up represented the second category. The score of each item was around 2 points, indicating that older people in this category have only about 2 family members or friends to interact with. We named it the social isolation risk category (SIRC, 30.45 and 33.57% at two time points). The scores of the third and fifth categories, represented by the top two lines, fluctuated around 3 and 4 points respectively, indicating that they have 3–4 or 5–8 family members or friends to interact with. We named them the medium-level social network category (MSNC, 49.18 and 45.32% at two time points) and the high-level social network category (HSNC, 8.91 and 7.10% at two time points), respectively. The fourth category was special; the average scores of items 1, 2, and 3 were close to 3 points, while the average scores of items 4, 5, and 6 were less than 1 point, indicating that older people in this category can interact with more family members in contacting, talking about personal matters, or seeking help, and almost no friends to interact with. We named it the friend-type social isolation category (FSIC, 4.90 and 4.57% at two time points; Table 2).

**Table 2:** Fitting indicators of LPA models of different potential categories at T1 and T2.

Time	Latent profiles	AIC	BIC	aBIC	Entropy	LMR-LRT(*p*)	BLRT(*p*)
T1 (2016)	1	136016.053	136098.632	136060.499			
	2	124693.719	124824.469	124764.091	0.804	< 0.001	< 0.001
	3	120394.936	120394.936	120312.313	0.835	< 0.001	< 0.001
	4	118325.265	118552.356	118447.489	0.831	< 0.001	< 0.001
	5	116470.545	116745.807	116618.696	0.840	< 0.001	< 0.001
	6	115707.075	116030.508	115881.152	0.826	< 0.001	< 0.001
T2 (2018)	1	134988.305	135070.884	135032.75			
	2	122459.189	122589.938	122529.561	0.822	< 0.001	< 0.001
	3	119318.955	119497.876	119415.254	0.770	< 0.001	< 0.001
	4	117511.627	117738.719	117633.852	0.822	< 0.001	< 0.001
	5	115492.199	115767.462	115640.351	0.845	< 0.001	< 0.001
	6	114699.915	115023.348	114873.993	0.857	< 0.001	< 0.001

AIC, Akaike Information Criteria. BIC, Bayesian Information Criteria. aBIC, Sample-Size Adjusted BIC. LMR-LRT, Vuong-Lo–Mendell–Rubin Likelihood Ratio Test. BLRT: Bootstrapped Likelihood Ratio Test.

### 3.2 Transitions of latent profiles of social isolation of older people

Without adding any covariates, the latent transition model was used to analyze the changes of five social isolation profiles from T1 to T2. The results are shown in Table 3. The diagonal of the transition matrix represents the probability that the subject will remain in the original potential state at two time points. The Entropy value of the model was 0.866, which demonstrated that the classification accuracy was good (57). The first 4 categories had high probability of keeping in the original groups from 2016 to 2018: 58.8, 68.8, 61.7, and 63.1%, respectively. The probability of keeping in HSNC was lower, 34.7%. As time passes, the older people of HSNC may translate into MSNC and SIRC, with 39.7 and 22.3% probability, respectively. The older people in FSIC may translate into MSNC with a 22.7% probability. The older people in MSNC may translate into SIRC with a 24.1% probability. The older people in SIRC may translate into MSNC with a 21.8% probability. The older people in SIC may translate into SIRC and MSNC with 15.3 and 19.2% probability, respectively.

**Table 3:** Latent transition probabilities from T1 (2016) to T2 (2018) based on the estimated latent transition model.

		T2 (2018)				
		SIC	SIRC	MSNC	FSIC	HSNC
T1 (2016)	SIC	**0.588**	0.153	0.192	0.035	0.032
	SIRC	0.042	**0.687**	0.218	0.013	0.040
	MSNC	0.059	0.241	**0.617**	0.024	0.059
	FSIC	0.032	0.084	0.227	**0.631**	0.026
	HSNC	0.011	0.223	0.397	0.022	**0.347**

SIC, SIRC, MSNC, FSIC, and HSNC were social isolation category, social isolation risk category, medium-level social network category, friend-type social isolation category, and high-level social network category, respectively. The bold value indicated the probabilities of remaining in the original categories from T1 to T2.

### 3.3 Influencing factors of social isolation of older people

#### 3.3.1 The effect of demographic variables on the grouping of social isolation

To explore the effects of gender, marital status, and household registration location on social isolation grouping of the older adults, multiple logistic regression analysis was carried out with SIC as the reference group, and the change ratio of the probability (Odds Ratio, OR) of the older adults belonging to other groups under the influence of covariates was obtained. In this study, gender (0 = male, 1 = female), household registration location (0 = rural, 1 = urban), and marital status (0 = widowed, 1 = non-widowed) were all classified variables. Male, agricultural household registration, and widowed older people were taken as reference groups, respectively, and which group female, urban household registration, and non-widowed older people were more likely to belong to was analyzed.

The results at T1 were shown in Table 4. Compared with males, females were more likely to be in SIRC than in SIC (OR = 1.292). Compared with the rural older people, the probabilities of the urban older people in SIRC (OR = 1.654) and in MSNC (OR = 1.339) were higher than that in SIC. Compared with widowed older people, non-widowed older people were more likely to be in SIC (OR = 0.576) than in FSIC.

**Table 4:** Odd ratios of latent state probabilities at T1 (2016) under the influence of covariates.

	SIRCvsSIC		MSNCvsSIC		FSICvsSIC		HSNCvsSIC	
	*β*	*OR*	*β*	*OR*	*β*	*OR*	*β*	*OR*
Gender	**0.256** ^*^	**1.292**	0.192	1.212	0.171	1.186	0.039	1.040
Household registration	**0.503** ^***^	**1.654**	**0.292** ^*^	**1.339**	0.238	1.269	−0.061	0.941
Marital status	0.135	1.145	−0.048	0.953	**−0.552** ^**^	**0.576**	0.126	1.123
Daily living activity ability	**0.158** ^***^	**1.171**	**0.223** ^***^	**1.250**	**−0.091** ^*^	**0.913**	**0.120** ^*^	**1.127**
Cognitive ability	**0.198** ^**^	**1.219**	**0.186** ^***^	**1.204**	−0.037	0.964	**0.456** ^***^	**1.158**

SIC, SIRC, MSNC, FSIC, and HSNC were social isolation category, social isolation risk category, medium-level social network category, friend-type social isolation category, and high-level social network category, respectively. β, standardized regression coefficient. OR, Odds Ratio. *p < 0.05, **p < 0.01, ***p < 0.001. Bold values indicated that the covariates had a statistically significant effect on the latent state probability.

The results at T2 were shown in Table 5. There was no significant difference in grouping possibilities between males and females. Compared with the rural older people, the probabilities of the urban older people in SIRC (OR = 1.448), MSNC (OR = 1.716), and HSNC (OR = 1.464) were higher than that in SIC. Compared with widowed older people, non-widowed older people were more likely to be in HSNC than in SIC (OR = 1.443).

**Table 5:** Odd ratios of latent state probabilities at T2 (2018) under the influence of covariates.

	SIRCvsSIC		MSNCvsSIC		FSICvsSIC		HSNCvsSIC	
	*β*	*OR*	*β*	*OR*	*β*	*OR*	*β*	*OR*
Gender	0.112	1.119	0.168	1.183	0.219	1.245	−0.062	0.940
Household registration	**0.370** ^***^	**1.448**	**0.540** ^***^	**1.716**	0.020	1.020	**0.381** ^*^	**1.464**
Marital status	0.195	1.215	0.209	1.232	−0.112	0.894	**0.367** ^*^	**1.443**
Daily living activity ability	**0.123** ^**^	**1.131**	**0.142** ^***^	**1.153**	**0.093** ^***^	**0.911**	**0.152** ^*^	**1.164**
Cognitive ability	0.035	1.036	0.196	1.101	**−0.314** ^***^	**0.731**	**0.191** ^*^	**1.210**

SIC, SIRC, MSNC, FSIC, and HSNC were social isolation category, social isolation risk category, medium-level social network category, friend-type social isolation category, and high-level social network category, respectively. β, standardized regression coefficient. OR, Odds Ratio. *p < 0 0.05, **p < 0 0.01, ***p < 0.001. Bold values indicated that the covariates had a statistically significant effect on the latent state probability.

#### 3.3.2 The effect of daily living activity ability and cognitive ability on the grouping of social isolation

We adopted multiple logistic regression analysis to explore the influence of aging factors on social isolation grouping, including daily living activity ability and cognitive ability.

The results on T1 were shown in Table 4. Older people with high daily living activity ability were more likely to be in SIRC (OR = 1.171), MSNC (OR = 1.250), and HSNC (OR = 1.127), and were less likely to be in FSIC (OR = 0.913), compared with in SIC.

Compared with belonging to SIC, older people with high cognitive ability were more likely to belong to SIRC (OR = 1.219), MSNC (OR = 1.204), and HSNC (OR = 1.158).

The results at T2 were shown in Table 5. Compared with belonging to SIC, the older people with high daily living activity ability were more likely to belong to SIRC (OR = 1.131), MSNC (OR = 1.153), FSIC (OR = 0.911), and HSNC (OR = 1.164).

Compared with belonging to SIC, the older people with high cognitive ability were more likely to belong to HSNC (OR = 1.210) and less likely to belong to FSIC (OR = 0.731).

#### 3.3.3 The effect of daily living activity ability and cognitive ability on the latent transition of social isolation profiles

The results of multiple regression analysis showed that gender, residence registration, marital status, daily living ability and cognitive ability had different effects on the classification and transformation of social isolation in the older adults.

Based on exploring the influence of various factors on the classification of social isolation of the older adults, we further explored the influence of various factors on the transition of social isolation categories of the older adults. The analysis results of the transition trend were shown in Table 6. From T1 to T2, for the older people with high daily living activity ability: compared with staying in SIC, the older people were less likely to transfer to MSNC (OR = 0.899) and FSIC (OR = 0.824); compared with staying in SIRC, the older people were less likely to change to FSIC (OR = 0.846); compared with staying in MSNC, the older people were more likely to transfer to SIC (OR = 1.113); compared with staying in FSIC, the older people were more likely to change to SIC (OR = 1.213) and SIRC (OR = 1.182).

**Table 6:** Odd ratios of transition probabilities under the influence of covariates.

Influence factors	Latent profiles	SIC	SIRC	MSNC	FSIC	HSNC
Daily living activity ability	SIC	REF	0.974	**0.899** ^*^	**0.824** ^*^	0.938
	SIRC	1.027	REF	0.923	**0.846** ^*^	0.963
	MSNC	**1.113** ^*^	1.084	REF	0.917	1.044
	FSIC	**1.213** ^*^	**1.182** ^*^	1.090	REF	1.138
	HSNC	1.066	1.038	0.958	0.878	REF
Cognitive ability	SIC	REF	**1.176** ^*^	**1.402** ^***^	1.109	**1.317** ^***^
	SIRC	**0.850** ^*^	REF	**1.192** ^***^	0.943	1.120
	MSNC	**0.713** ^***^	**0.839** ^***^	REF	**0.791** ^**^	0.940
	FSIC	0.901	1.060	**1.264** ^**^	REF	1.187
	HSNC	**0.759** ^***^	0.893	1.064	0.842	REF

SIC-HSNC were social isolation category, social isolation risk category, medium-level social network category, friend-type social isolation category, and high-level social network category, respectively. *p < 0.05, **p < 0.01, ***p < 0.001. Bold values indicated that the influence of covariates on the probability of state transition were statistically significant.

From T1 to T2, for the older people with high cognitive ability: compared with staying in SIC, the older people were more likely to transfer to SIRC (OR = 1.176), the MSNC (OR = 1.402), and HSNC (OR = 1.317); compared with staying in SIRC, the older people were more likely to transfer to MSNC (OR = 1.192) and less likely to transfer to SIC (OR = 0.850); compared with staying in MSNC, the older people were less likely to change to SIC (OR = 0.713), SIRC (OR = 0.839), and FSIC (OR = 0.791); compared with staying in FSIC, the older people were more likely to transfer to MSNC (OR = 1.264); compared with staying in HSNC, the older people were less likely to change to SIC (OR = 0.759).

## 4 Discussion

In this study, latent profile analysis and latent transition analysis were used to analyze the latent categories of social isolation of older people and their change patterns over time. Logistic regression analysis was used to explore the effects of demographic variables, daily living ability, and cognitive ability on the classification of social isolation, as well as the effects of daily living ability and cognitive ability on the transformation of social isolation of the Chinese older people.

### 4.1 Heterogeneity of social isolation for older people

Our study results proved that there was great heterogeneity in the social isolation states among Chinese older people, which verified hypothesis 1. The medium-level social network category had the largest population, and the next was the social isolation risk category. The population of the social isolation category and the high-level social network category were slightly different at the two time points. There was a special category for older people—the friend-type social isolation category compared with some studies (58, 59). The difference may be attributed to the measurement tools used. Another study, comparing average scores, finds that older people are more isolated from friends than from family (56), supporting the validity of this classification to some extent. Their scores on the two dimensions of family network and friend network were significantly different. It indicated that family and friends had different effects on the social isolation of older people. China’s traditional culture attaches special importance to the family concept. The Confucian socio-ethical vision “Filial piety is the first” made family support still the main way for older people in China (60). A survey finds that 85.4% of older people are willing to select home-based old-age care (61). When older people are ill or incapacitated, they are more likely to seek help from family members and decrease participation in social activities, increasing the risk of friend isolation. So, the social aspect should build perfect social places to increase social chances for older people. The family aspect should encourage and support older people to engage in social activities with friends.

### 4.2 Transitions of latent categories of social isolation for older people

Developmental situation theory suggests that individual development and change are unique, influenced by historical time, life stages, and critical events (21). Although for SIC, SIRC and MSNC categories, the probabilities of remaining in the original categories were relatively large, there were still about 40% of the older adults will change to other categories. More than 60% of those in the HSNC category moved toward worse social isolation categories. These findings support hypothesis 2.

Although the social isolation status of the older adults changed between the two time points, the MSNC and SIRC categories were the largest at both time points. At the same time, the stability of remaining in the MSNC and SIRC categories over time was relatively high, except that the high-level social network category was more likely to change to the medium-level social network category and the social isolation risk category. It suggested that once social isolation in older people was formed, it was difficult to alleviate by itself, and there was a risk of deterioration. Therefore, it was crucial to carry out targeted identification and intervention in the early stage when older people enter different degrees of social isolation. Although the transition from social isolation to a high level of social network status was rare, we found that a small number of older people made a transition to a relatively good social network status. This illustrated the dynamic nature of social isolation and once again suggests that we must take targeted measures based on the heterogeneity of social isolation in older people (19). Previous studies have found patterns of growth or stability of social networks in older people (62, 63), which may be the focus of future work of community workers and family caregivers.

### 4.3 Influencing factors of latent profiles and latent transition of social isolation for older people

The results of multiple regression analysis showed that gender, household registration, marital status, daily living ability and cognitive ability had varying effects on the classification and transition of social isolation status in the older adults, which verified research hypothesis 3. Influences of the demographic variables on latent profiles of social isolation were slightly different, but the overall trend was similar at T1 and T2. There was almost no significant difference in the likelihood between male and female older people belonging to the social isolation category and other categories. The previous contradictory research results may be due to the sample size or geographical reasons. For example, a study in Nigeria finds that widowed women are more likely to experience loneliness in later life, which is associated with local polygamy (64). Compared with the rural older people and the widowed older people, the urban older people and the non-widowed older people, respectively, showed a lower possibility of belonging to the social isolation category. It meant that the rural older people and the widowed older people have a greater risk of social isolation (65, 66).

There was an overall trend: the older people with high daily living activity ability and cognitive ability were less likely to belong to the social isolation category at T1 and T2. Older people might withdraw from social life passively because of their deteriorating health (67). So, we should pay more attention to the rural, widowed, low daily living activity ability, and low cognitive ability older people and increase their social chances and social convenience.

Both the daily living activity ability and cognitive ability are important indicators of measuring the function of older people (68, 69). They are closely related to social isolation, too. We found that they affect the transition of social isolation in diverse ways. Compared with remaining in the original category, the older people with high daily living activity ability were more likely to change to the more serious social isolation category and less likely to move to the better social network category from T1 to T2. This seemed contrary to the previous conclusion that the greater the daily living activity ability, the less likely social isolation was. However, this shifting trend illustrates the likelihood of changes in the social isolation category over time, not the relationship between the two at a time point. Maybe the reason was that older people with high daily living activity ability were less likely to need active care and help, leading to the neglect of their social communication needs by family and friends. So, older people with high daily living activity ability had a greater possibility of changing into a more serious social isolation category as time went on. It indicated that we should also pay attention to changes in the social interaction status of older people with high daily living activity ability; otherwise, it would be possible to change to a more serious social isolation group.

The older people with high cognitive ability showed an overall trend toward better social network categories compared with remaining in the original category from T1 to T2. Older people with strong cognitive ability could fully understand and use social network relationships, maintain a good social network state, and even change to a better direction. This also suggested that people should pay special attention to the protection and strengthening of older people’s cognitive ability when taking care of older people, such as carrying out effective ability training. If the older adults were physically allowed, they should insist on exercise, undertake life affairs within their capacity, use their brains appropriately, and maintain good cognitive function.

## 5 Conclusion and limitations

### 5.1 Conclusion

Social isolation of older people in China could be divided into five latent profiles: social isolation category, social isolation risk category, medium-level social network category, friend-type social isolation category, and high-level social network category. Rural older people and widowed older people were more likely to belong to the social isolation category compared with urban older people and non-widowed older people. Older people with high daily living activity ability and cognitive ability were less likely to belong to the social isolation category. As time went on, the social isolation of older people had a strong stability, but older people in the high-level social network category had the risk of changing to the medium-level social network category and social isolation risk category. With the development of time, older people with high daily living activity ability were more likely to change to the more serious social isolation categories, and older people with strong cognitive ability were more likely to change to the better social isolation categories.

### 5.2 Limitations

First, only 2 waves of data separated by 2 years were used for the transition analysis of social isolation. Future studies need to explore the shifting patterns of social isolation with more wave data collection over a longer period to draw more stable and reliable conclusions. Second, the measurement indexes in this study were not rich enough: the number of family members or friends to contact, talk about personal matters, or seek help. Future studies may consider including the social networks between older people and community workers, work partners, community health care workers, etc., and consider the quality indicators of social networks. Third, the data we used was from CLASS, but it was gathered before the COVID-19 pandemic, and the pandemic changed people’s lifestyles and social networks. Therefore, further investigation of changes in the social isolation of older people during and after the pandemic will be necessary.

## Data Availability

The data of this study came from the China Longitudinal Aging Social Survey, CLASS (http://class.ruc.edu.cn/). The data are available from the corresponding author upon reasonable request.
